# Monitoring Oxygenation and Gas Exchange in Neonatal Intensive Care Units: Current Practice in the Netherlands

**DOI:** 10.3389/fped.2015.00094

**Published:** 2015-11-03

**Authors:** Ratna N. G. B. Tan, Estelle E. M. Mulder, Enrico Lopriore, Arjan B. te Pas

**Affiliations:** ^1^Department of Pediatrics, Division of Neonatology, Leiden University Medical Center, Leiden, Netherlands

**Keywords:** questionnaire, oxygenation monitoring, gas exchange monitoring, neonatal intensive care unit, current practice

## Abstract

**Background:**

Although recommendations in oxygenation and gas exchange monitoring in the neonatal intensive care unit (NICU) are available, little is known of the current practice.

**Aim:**

To evaluate the current practice in oxygenation and gas exchange monitoring of the NICUs in the Netherlands.

**Methods:**

An online survey-based questionnaire concerning preferences and current practice of monitoring oxygenation and gas exchange was sent out to all 107 neonatal staff members (neonatologists, neonatal fellows, and physician assistants) of the 10 NICUs in the Netherlands.

**Results:**

The response rate was 42%. Pulse oximetry (PO), partial pressure of oxygen in arterial blood gas (paO_2_), and oxygen saturation in arterial blood gas (saO_2_) was used by, respectively, 100, 80, and 27% of the staff members for monitoring oxygenation. Of all staff members, 76% considered PO as the best parameter for monitoring oxygenation, 22% paO_2_, and 2% saO_2_. Blood gas, transcutaneous gas monitoring, endotracheal gas monitoring, and near-infrared spectroscopy was used by, respectively, 100, 82, 40, and 18% of the staff members for monitoring gas exchange. During endotracheal ventilation, 67% of the caregivers would exclusively accept arterial blood gas for gas exchange monitoring. In contrast, during non-invasive ventilation, 68% of the caregivers did not prefer arterial or capillary blood gas (CBG). CBG is found reliable in infants with warm extremities by 76% of the caregivers. Venous blood gas would be accepted by 60% of the caregivers, independent of the mode of respiratory support, and only when venous blood sample was needed for other reasons.

**Conclusion:**

This survey identified a wide variation in preference in monitoring oxygenation and gas exchange monitoring among Dutch neonatal staff members.

## Introduction

Monitoring of gas exchange and oxygenation is daily practice in infants admitted in the neonatal intensive care unit (NICU). Several methods for monitoring are available and several studies compared invasive and non-invasive methods: arterial blood gas (ABG), capillary blood gas (CBG), and venous blood gas (VBG) analyses (1–6), pulse oximetry (PO) (7–11), transcutaneous (tc) gas monitoring (i.e., tcO_2_, tcCO_2_) (9, 12–17), end-tidal gas monitoring (etCO_2_) (18–21), and near-infrared spectroscopy (NIRS) (22).

The ABG is considered the gold standard, but there is 10–25% failure of placing of an arterial line and also severe complication (as limb ischemia, necrosis) can occur (1, 4, 22, 23). The best alternative is the CBG in a well-perfused infant (1, 2, 4–6, 24–26). However, VBG is not recommended because various (small) studies show conflicting results (1, 3, 6, 23, 27, 28). Transcutaneous O_2_ and CO_2_ are considered the best non-invasive method; studies have shown that the values correlate well with ABG values (9, 12–17). PO has become standard of care to monitor oxygenation in the NICU; it is easy to use, and monitoring is continuous. However, studies showed that saturation of PO is not interchangeable with saturation in ABG (7, 9, 11, 29, 30). End-tidal CO_2_ measurement is only recommended for confirming endotracheal tube position (18–21, 31, 32).

Although all studies reported the advantages and disadvantages of the different methods and methods are recommended, the choice of the caregiver on what to use in daily practice is also influenced on the availability, practicality, and local preferences. Guidelines are needed to aim for a uniform policy, but first it is important to know how the current daily practice corresponds with the available recommendations. To investigate this, we performed a survey to inventory the current practice in monitoring oxygenation and gas exchange in all NICUs in the Netherlands.

## Materials and Methods

We sent a questionnaire to the 107 neonatal staff members [84 neonatologists, 18 neonatal fellows, and 5 physician assistants/nurse practitioners (PA/NP)] working in the 10 Dutch tertiary NICU centers in 2012, who are registered by the Dutch Association of Pediatrics. The questionnaire included questions concerning the daily practice in monitoring oxygenation and gas exchange (see Table 1). The units in the Netherlands are all up-to-date level III NICUs, and there are very little difference in resources and employees. For this reason, we aimed for a national overview. Most equipment for monitoring is available in all Dutch NICUs. We aimed for at least three responders per NICU, a first general reminder was sent after 4 weeks, and the last reminder was sent specific to NICUs when the response rate was less than three. The local institutional ethical review board approved the questionnaire study.

**Table 1:** **Questionnaire Dutch neonatologists**.

Category	Question	
Methods and interpretation of monitoring oxygenation	1	Which measurement do you use to monitor the oxygenation status of the infant? (pulse oximetry, saturation in blood gas, paO_2_, NIRS, cvsO_2_ and tcO_2_)
	2	What do you find the best parameter to monitor oxygenation status? (pulse oximetry, saturation in blood gas, paO_2_, NIRS, and tcO_2_)
	3	Does your blood gas analyzer have a co-oximetry unit? (yes, no, I do not know what a co-oximetry unit is)
Methods and interpretation of monitoring gas exchange	4	Which measurement do you use to monitor gas exchange of the infant? (paCO_2_, end-tidal CO_2_, tcO_2_, and NIRS)
	5	What do you find the best parameter to monitor gas exchange? (paCO_2_, end-tidal CO_2_, tcO_2_, and NIRS)
Interpretation of arterial, venous, and capillary blood gases	6	Does ventilation modus (invasive and non-invasive ventilated) matter for preference for type of blood gas (ABG, CBG, and VBG)?
	7	Which component of the analysis of the blood gas (CBG and VBG) is reliable? (pH, pCO_2_, pO_2_, base excess, sodium bicarbonate, and oxygen saturation)
	8	On what condition is the reliability of a CBG dependent? (warm/pink extremities, capillary refill time, central core temperature, and independent of circulatory status of the infant)
	9	Do you accept a VBG when VENOUS blood is needed, in an infant with respiration support WITHOUT ARTERIAL access and a blood gas sample is needed?

*PO, pulse oximetry; paO_2_, partial pressure of oxygen in arterial blood gas; tcO_2_, transcutaneous O_2_; NIRS, near-infrared spectroscopy; cvsO_2_, central venous saturation; paCO_2_, partial pressure of carbon dioxide in arterial blood gas; tcCO_2_, transcutaneous CO_2_; etCO_2_, end-tidal CO_2_*.

### Statistical Analysis

All replies were collected in MS Excel 2010 and expressed as percentages of total participants.

## Results

From the 10 NICU centers, 45/107 (42%) neonatal staff members responded and returned the questionnaire; of which 35 (42%) neonatologists, 8(44%) neonatal fellows, and 2(40%) PA/NP. From each unit at least three staff members responded. All staff members indicated that there were no specific guidelines available in the unit concerning monitoring oxygenation and gas exchange.

### Oxygenation

To monitor oxygenation, 100% of the staff members used PO, 80% used partial pressure of oxygen in ABG (paO_2_), and 27% oxygen saturation in ABG (saO_2_); while tcO_2_ (7%), NIRS (2%), lactate (2%), and central venous saturation oxygenation (2%) were rarely used (Table 2). Most staff members (76%) considered PO the best parameter for monitoring oxygenation status, 22% paO_2_, and 2% saO_2_. The staff members using saO_2_, 75% of them were not aware whether co-oximetry measures saO_2_, or estimates it, based on paO_2_. While most staff members in all units use PO and paO_2_, the use of saO_2_, tcO_2_, NIRS, and lactate was variable in and between units (Table 2).

**Table 2:** **Oxygenation monitoring in the NICU**.

%^a^	PO	paO_2_	saO_2_	tcO_2_	NIRS	Lactate	cvsO_2_
NICU 1	100	67	17				
NICU 2	100	100				25	25
NICU 3	100	75		50			
NICU 4	100	78	33	11			
NICU 5	100	100	33				
NICU 6	100						
NICU 7	100	100	4		20		
NICU 8	100	100	67				33
NICU 9	100	100		33			
NICU 10	100	100	25				
All NICUs	100	80	27	7	2	2	2
All NICUs and considered the best	76	22	2				

*PO, pulse oximetry; paO_2_, partial pressure of oxygen in arterial blood gas; tcO_2_, transcutaneous O_2_; NIRS, near-infrared spectroscopy; cvsO_2_, central venous saturation*.

*^a^Parameter used in percentage*.

### Gas Exchange

To monitor gas exchange, 100% of the staff members used paCO_2_, 82% tcCO_2_, 40% etCO_2_, 18% NIRS, and 7% tidal volume (Table 3). Most staff members (91%) considered paCO_2_ the best parameter for monitoring gas exchange, in contrast to tcCO_2_ (9%). The use of tcCO_2_ was very variable in and between units (Table 4).

**Table 3:** **Gas exchange monitoring in the NICU**.

%^a^	paCO_2_	tcCO_2_	etCO_2_	NIRS	Tidal volume
NICU 1	100	100			
NICU 2	100	100	75	50	
NICU 3	100	100	75		
NICU 4	100	78			
NICU 5	100	100	100		
NICU 6	100	100			
NICU 7	100	80	60	80	40
NICU 8	100	67	100		
NICU 9	100	100	100	66	
NICU 10	100				
All NICUs	100	82	40	18	7
All NICUs and considered the best	91	9			

*paCO_2_, partial pressure of carbon dioxide in arterial blood gas; tcCO_2_, transcutaneous CO_2_; etCO_2_, end-tidal CO_2_; NIRS, near-infrared spectroscopy*.

*^a^Parameter used in percentage*.

**Table 4:** **Transcutaneous gas monitoring and reliability, according to NICU members**.

%^a^	(Almost) never	As trend monitoring	Depend on the patient	In most of the patients	In most of the prematures
NICU 1	0	0	17	33	50
NICU 2	0	0	50	0	50
NICU 3	0	50	50	0	0
NICU 4	11	11	22	22	33
NICU 5	0	0	33	33	33
NICU 6	0	0	0	50	50
NICU 7	0	0	0	0	100
NICU 8	0	0	0	33	67
NICU 9	0	33	33	0	33
NICU 10	25	25	25	25	0
All NICUs	4	11	22	20	42

*^a^Percentage of respondents*.

### Blood Gas

**Figure 1:**
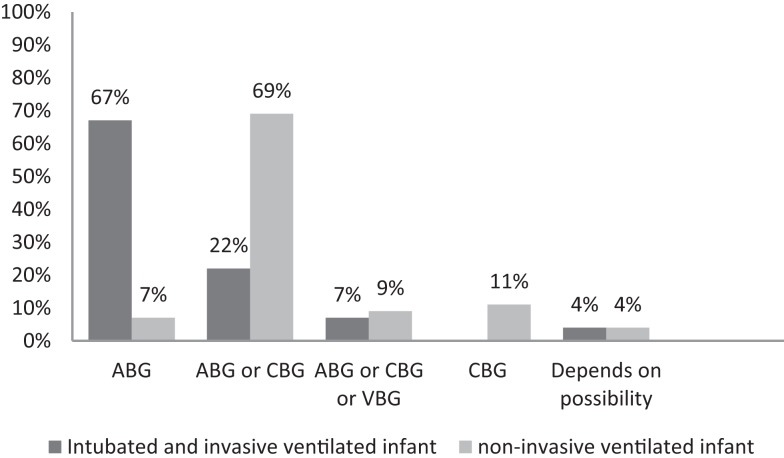
**Percentage used blood gas in an invasive and non-invasive ventilated patients**. ABG, arterial blood gas; CBG, capillary blood gas; VBG, venous blood gas.

The type of blood gas (ABG, CBG, or VBG) used depended on the ventilation modus of the infant (Figure 1). In all units, most often ABG is used in intubated and mechanically ventilated infants, while in non-invasive ventilated infants, both ABG and CBG were preferential. Staff members considered the reliability of CBG depended on the presence of warm extremities (76%), capillary refill time <2 s (44%), pink extremities (37%), and normal central body temperature (22%). Overall, staff members considered CBG more reliable than VBG (Table 5); nevertheless, 11% uses VBG in ventilated infants and 13% in non-invasive ventilated infants (Figure 1). VBG and CBG were not considered reliable at all by 13 and 4% of the caregivers, respectively (Table 5). In contrast, this opinion changes when a blood gas was due when venous blood sampling was simultaneously needed; the majority (60%) of the clinicians would then accept a VBG.

**Table 5:** **Reliability of different parts of the capillary (CBG) and venous blood gas (VBG)**.

%^a^	CBG	VBG
pH	96	78
pCO_2_	87	51
Sodium bicarbonate	76	44
Base excess	78	49
pO_2_	9	4
Oxygenation saturation	4	4
None of the above	4	13

*^a^Parameter used in percentage*.

## Discussion

This study provided an overview of the current clinical practice. Although most recommendations concerning monitoring gas exchange are followed, there is a variance in and between units in opinion about reliability and interpretation of the measurements. Oxygenation and gas exchange were most often monitored by PO, tcCO_2_, and ABG, while other current available measurements are infrequently or rarely used. Factors like practicality, personal experiences and aiming for less cumbersome measurements probably influenced current practice in monitoring.

The response rate was low (42%), but this is higher than the average response rate of online voluntary survey in physician specialists (33). We anticipated a low response, and we therefore aimed for at least three responders of each NICU. We reached this target with a mix of inexperienced and experienced users. We believe that the respondents give a good reflection of the Dutch neonatal staff members.

According to the literature, the best method for monitoring hypoxia and hyperoxia is an ABG (analyzed with a co-oximetry unit), but we observed that staff members infrequently used the oxygen saturation in the ABG (27%). In addition, most caregivers were not aware whether the blood gas analyzer uses co-oximetry or is an estimation. While co-oximetry would be more accurate than PO; caregivers considered PO as the best and most commonly used monitoring for oxygenation. PO is non-invasive, non-cumbersome, easy to apply, and will provide continuous measurements. However, oxygen saturation can be overestimated by PO (7) and is inaccurate at oxygen saturation levels <70% and in hyperoxia >95% (29, 34). The other disadvantages of the PO are motion artifacts, even in the motion resistant types, and low perfusion quality disturbances depending on probe site placement (29).

While studies recommended continuous tcO_2_ monitoring for preventing hypoxemia/hyperoxemia (15, 32), few caregivers (7%) used this. In contrast, most caregivers considered tcCO_2_ the best non-invasive continuous monitoring of CO_2_, which is consistent with a recent German survey (35). Studies demonstrated that tcO_2_/CO_2_ is well correlated with paO_2_/CO_2_, but tcO_2_ often underestimated paO_2_ while tcCO_2_ overestimated paCO_2_ (12, 15–17, 32, 35–37). In contrast to the literature, most caregivers considered tc measurements not reliable and is possibly mostly used for trend monitoring. It is possible that the frequency of replacement and calibrations has influenced the caregiver’s experience in the reliability. While in the performed studies, the sensor is frequently replaced and calibrated, in daily practice this is not always possible. In addition, dislocated adhesive rings, skin burns, and decubitus can discourage caregiver in using tc monitoring. In this view, the preference of staff members to use tc monitoring in preterm infants was surprising, especially considering the fact that correlation is poor in preterm infants when compared to term infants (32, 38). End-tidal CO_2_ monitoring is not frequently used in the Netherlands, most probably because the inaccuracy due to high respiratory rate, the low-tidal volume, and the increase of dead space volume (19).

The staff members considered ABG analysis the gold standard for monitoring gas exchange, which is consistent with the literature (23, 31, 36). The preference of performing ABG in ventilated infants probably reflects that most of the ventilated infants have an arterial catheter for hemodynamic monitoring, while CBG is well accepted in non-invasive ventilated infants. The reliability of CBG can be poor by disturbed perfusion, which is more likely to occur in the more critical and mechanical ventilated infants. The staff members considered the CBG for pH, pCO_2_, and sodium bicarbonate in a child with warm extremities reliable, which is consistent with the current literature (1, 24, 26, 36).

The use of VBG is currently not recommended, as the studies performed are small and showed conflicting results (1, 3, 6, 23, 27, 28). Nevertheless, most staff members will accept a VBG when venous blood withdrawing will occur, most likely due to practical considerations. However, so far there is no data whether VBG and CBG can be interchangeably used. Gillivray et al. concluded that VBG would be useful for decision making in the need for intubation, but not for monitoring ventilation (6).

## Conclusion

This survey identified a large variation in oxygenation and gas exchange monitoring in the Dutch NICUs. Although guidelines are absent in the units, recommendations are followed combined with practical considerations. In current daily practice, PO is considered the best method of monitoring oxygenation and blood gasses for monitoring gas exchange. The preference for the type of blood gas analysis depends on circumstances: type of respiratory support and the opportunity of simultaneously venous blood withdrawing. Tc gas monitoring is then considered the best non-invasive measurement for gas exchange but is infrequently used. These results can be helpful when developing a guideline that is evidence based as well as practical.

## Conflict of Interest Statement

The authors declare that the research was conducted in the absence of any commercial or financial relationships that could be construed as a potential conflict of interest.
